# Author Correction: Tumour mutations in long noncoding RNAs enhance cell fitness

**DOI:** 10.1038/s41467-023-41288-5

**Published:** 2023-09-06

**Authors:** Roberta Esposito, Andrés Lanzós, Tina Uroda, Sunandini Ramnarayanan, Isabel Büchi, Taisia Polidori, Hugo Guillen-Ramirez, Ante Mihaljevic, Bernard Mefi Merlin, Lia Mela, Eugenio Zoni, Lusine Hovhannisyan, Finn McCluggage, Matúš Medo, Giulia Basile, Dominik F. Meise, Sandra Zwyssig, Corina Wenger, Kyriakos Schwarz, Adrienne Vancura, Núria Bosch-Guiteras, Álvaro Andrades, Ai Ming Tham, Michaela Roemmele, Pedro P. Medina, Adrian F. Ochsenbein, Carsten Riether, Marianna Kruithof-de Julio, Yitzhak Zimmer, Michaela Medová, Deborah Stroka, Archa Fox, Rory Johnson

**Affiliations:** 1 Department of Medical Oncology, Inselspital, Bern University Hospital, University of Bern, 3010 Bern, Switzerland; 2 Department for BioMedical Research, University of Bern, 3008 Bern, Switzerland; 3 Institute of Genetics and Biophysics “Adriano Buzzati-Traverso”, CNR, 80131 Naples, Italy; 4 Graduate School of Cellular and Biomedical Sciences, University of Bern, 3012 Bern, Switzerland; 5 School of Biology and Environmental Science, University College Dublin, Dublin, D04 V1W8 Ireland; 6 Conway Institute for Biomolecular and Biomedical Research, University College Dublin, Dublin, D04 V1W8 Ireland; 7 The SFI Centre for Research Training in Genomics Data Science, Dublin, Ireland; 8 Department of Visceral Surgery and Medicine, Inselspital, Bern University Hospital, University of Bern, Bern, Switzerland; 9 Department of Urology, Inselspital, Bern University Hospital, Bern, Switzerland; 10 Department of Radiation Oncology, Inselspital, Bern University Hospital and University of Bern, Bern, Switzerland; 11 School of Molecular Sciences, University of Western Australia, Crawley, WA Australia; 12 School of Human Sciences, University of Western Australia, Crawley, WA Australia; 13 GENYO, Centre for Genomics and Oncological Research, Pfizer/University of Granada/Andalusian Regional Government, Granada, 18016 Spain; 14 Instituto de Investigación Biosanitaria, Granada, 18014 Spain; 15 Department of Biochemistry and Molecular Biology I, University of Granada, Granada, 18071 Spain

**Keywords:** Long non-coding RNAs, CRISPR-Cas systems, Cancer genomics

Correction to: *Nature Communications* 10.1038/s41467-023-39160-7, published online 08 June 2023

The original version of this Article omitted from the author list the 25th author, Pedro P. Medina, who is affiliated with GENYO, Centre for Genomics and Oncological Research, Pfizer/University of Granada/Andalusian Regional Government, Granada, 18016, Spain, and Instituto de Investigación Biosanitaria, Granada, 18014, Spain, and Department of Biochemistry and Molecular Biology I, University of Granada, Granada, 18071, Spain. Consequently, his initials were added to the Author Contributions as having ‘supervised aspects of bioinformatic analysis’. This has been corrected in both the PDF and HTML versions of the article.

