# Supplementary material for: Tumour mutations in long noncoding RNAs enhance cell fitness
Source: Nat Commun. 2023 Jun 8;14:3342. doi: 10.1038/s41467-023-39160-7 (PMC10250536; doi:10.1038/s41467-023-39160-7)
Supplement: Supplementary file 1 — Supplementary Information [file 41467_2023_39160_MOESM1_ESM.pdf]

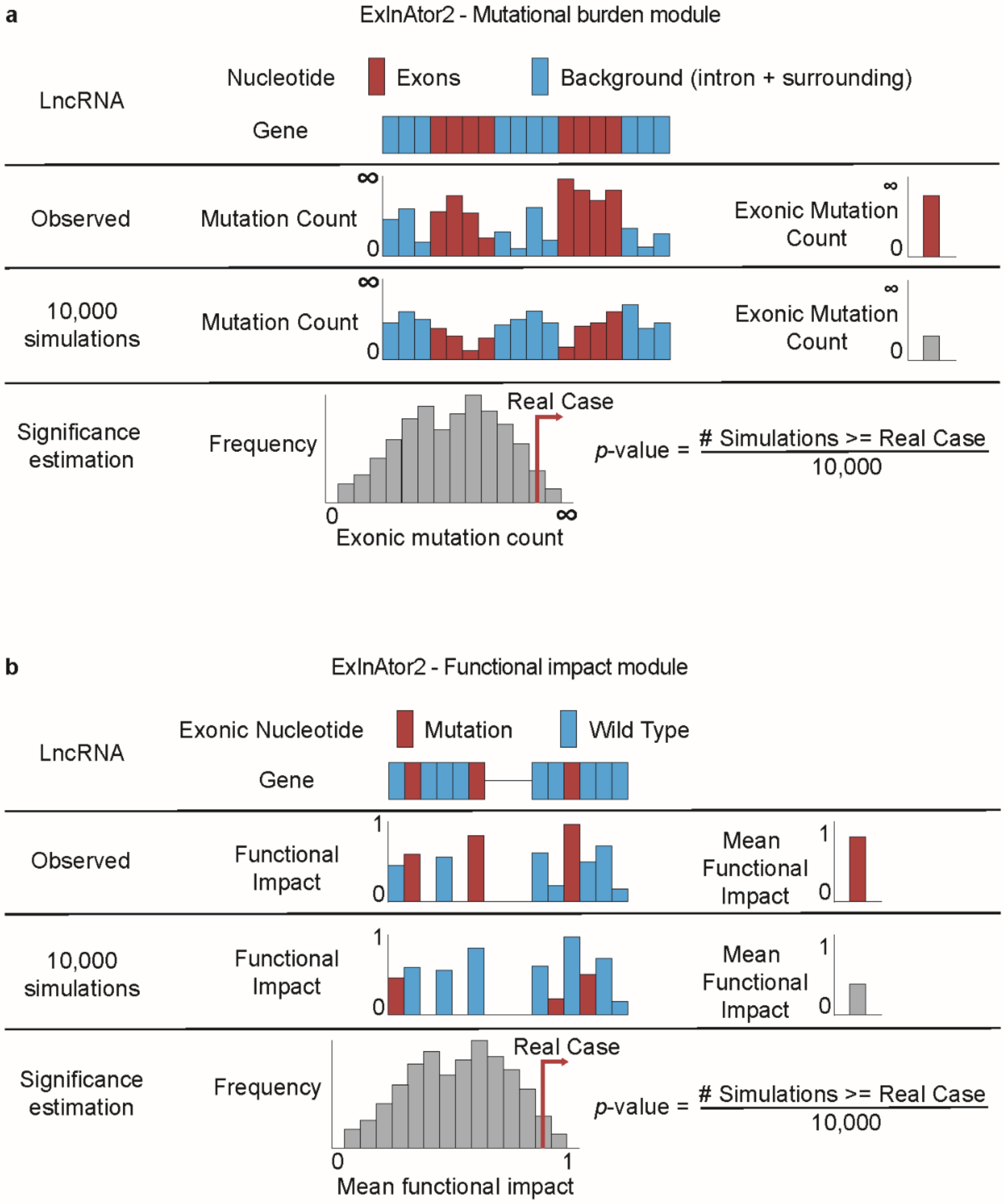
a
ExInAtor2 - Mutational burden module
LncRNA
Nucleotide
Exons
Background (intron + surrounding)
Gene
Observed
Mutation Count
∞
0
Exonic Mutation Count
10,000 simulations
Significance estimation
Frequency
Real Case
Exonic mutation count
p-value = # Simulations >= Real Case / 10,000
b
ExInAtor2 - Functional impact module
Exonic Nucleotide
Mutation
Wild Type
Functional Impact
1
Mean Functional Impact
Mean functional impact

Supplementary Figure 1

Overview of ExInAtor2 software

a) Graphic representation of the Mutational Burden module of ExInAtor2. Genes are divided in exons and background regions (including introns and flanking regions defined by the user). The number of real exonic mutations is calculated (‘observed’). Mutations in both exons and background are randomly shuffled, maintaining the overall trinucleotide content, a number of times indicated by the user (10,000 as example), and the number of exonic mutations in each iteration is recorded. The *p*-value is calculated by counting how many shuffled values are greater than or equal to observed number, divided by the total number of shuffles performed. **b)** Graphic representation of the Functional Impact module of ExInAtor2. Only exonic regions are considered. The mean functional impact score is calculated for all mutated positions (‘observed’). In a number of simulations defined by the user (10,000 as example), mutations are randomly shuffled along the exons while maintaining the trinucleotide content, and the mean functional impact score is recorded. The *p*-value is calculated by counting how many shuffled values are greater than or equal to the observed value, divided by the total number of shuffles. In both panels an empirical p-value (custom) is calculated on simulations (defined as higher or equal than observed value).

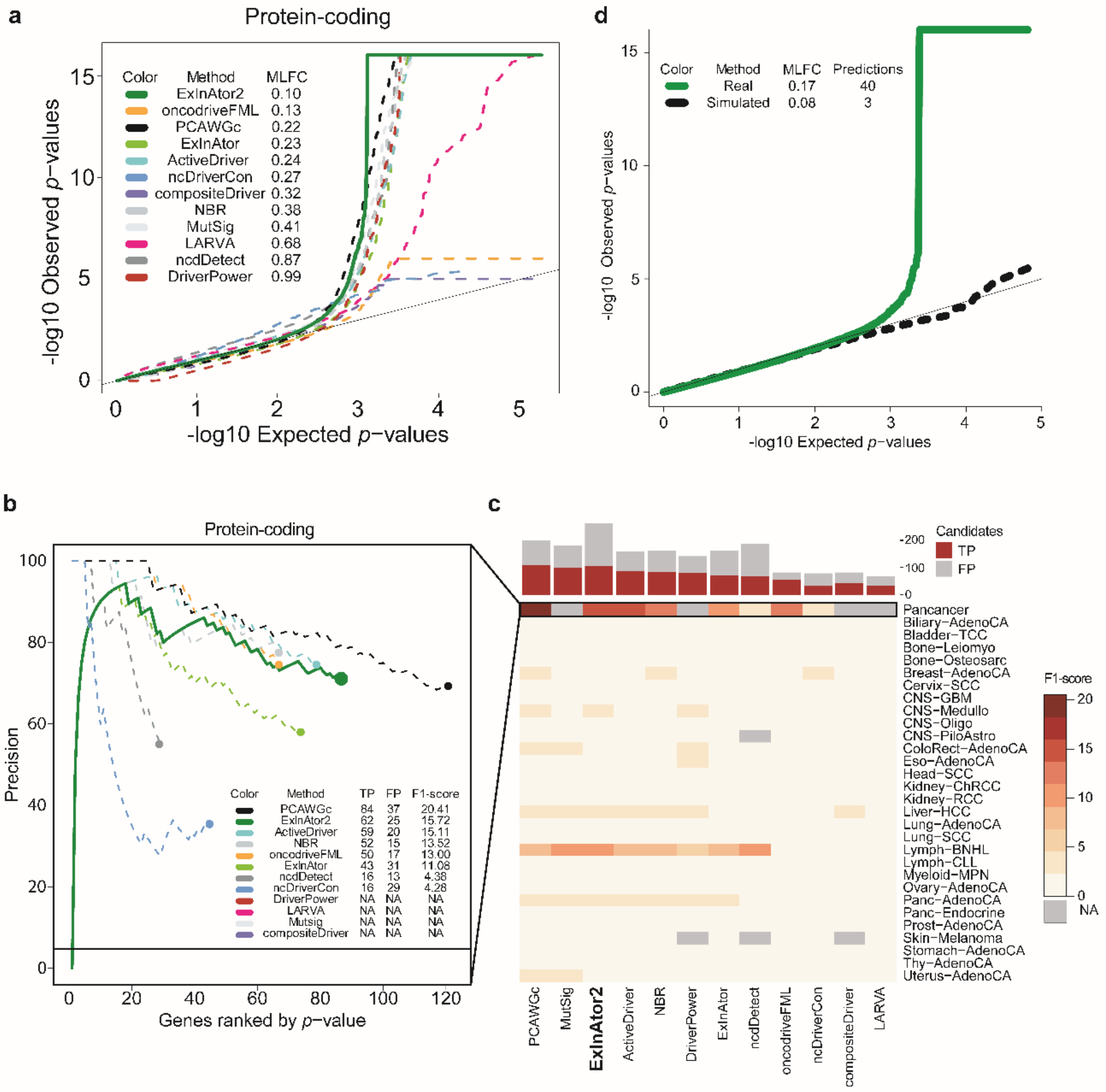

a
Protein-coding
Color Method MLFC
ExInAtor2 0.10
oncodriveFML 0.13
PCAWGc 0.22
ExInAtor 0.23
ActiveDriver 0.24
ncDriverCon 0.27
compositeDriver 0.32
NBR 0.38
MutSig 0.41
LARVA 0.68
ncdDetect 0.87
DriverPower 0.99
-log10 Observed p-values
-log10 Expected p-values
d
Color Method MLFC Predictions
Real 0.17 40
Simulated 0.08 3
-log10 Observed p-values
-log10 Expected p-values
b
Protein-coding
Precision
Genes ranked by p-value
Color Method TP FP F1-score
PCAWGc 84 37 20.41
ExInAtor2 62 25 15.72
ActiveDriver 59 20 15.11
NBR 52 15 13.52
oncodriveFML 50 17 13.00
ExInAtor 43 31 11.08
ncdDetect 16 13 4.38
ncDriverCon 16 29 4.28
DriverPower NA NA NA
LARVA NA NA NA
Mutsig NA NA NA
compositeDriver NA NA NA
c
Candidates
TP
FP
F1-score
NA
Pancancer
Biliary-AdenoCA
Bladder-TCC
Bone-Leiomyo
Bone-Osteosarc
Breast-AdenoCA
Cervix-SCC
CNS-GBM
CNS-Medullo
CNS-Oligo
CNS-PiloAstro
ColoRect-AdenoCA
Eso-AdenoCA
Head-SCC
Kidney-ChRCC
Kidney-RCC
Liver-HCC
Lung-AdenoCA
Lung-SCC
Lymph-BNHL
Lymph-CLL
Myeloid-MPN
Ovary-AdenoCA
Panc-AdenoCA
Panc-Endocrine
Prost-AdenoCA
Skin-Melanoma
Stomach-AdenoCA
Thy-AdenoCA
Uterus-AdenoCA
PCAWGc
MutSig
ExInAtor2
ActiveDriver
NBR
DriverPower
ExInAtor
ncdDetect
oncodriveFML
ncDriverCon
compositeDriver
LARVA

Supplementary Figure 2

ExInAtor2 displayed competitive performance

a) Evaluation of p-value distributions for driver prediction in protein-coding genes. Quantile-quantile plot (QQ-plot) shows the distribution of observed vs expected –log10 p-values (uncorrected for multiple hypothesis testing) from each method across all cohorts (as described in[1]). The Mean Log-Fold Change (MLFC) quantifies the difference between observed and expected values (Methods). **b)** Benchmark of driver discovery methods in PCAWG Pancancer dataset for protein-coding genes. x-axis represents genes sorted by increasing p-value (uncorrected for multiple hypothesis testing) for each method (as described in[1]). y-axis shows the percentage of true positives amongst cumulative set of candidates (precision) at each step of the x-axis (precision). Black horizon line shows the baseline, being the percentage of positives in the whole list of tested genes, ie the precision expected by random chance. Coloured dots represent the number of candidates for each method with adjusted p-value≤0.1 (Benjamini-Hochberg method). Table shows the number of True Positives (TP), False Positives (FP) and F1-score (Methods) for each driver method. **c)** Protein-coding gene benchmark for all PCAWG cohorts. Cells show the F1-score of each driver method (x-axis) in each cohort (y-axis). Grey cells correspond to cohorts where the method was not run. The bar plot at the top indicates the non-redundant total number of True Positives (TP) and False Positives (FP) unique candidates detected across all cohorts, i.e., if a gene is detected in multiple cohorts it is considered only once. Driver methods on the x-axis are sorted from left to right according to the F1-score of unique candidates. **d)** QQ plots displayed neutral behaviour (MLFC 0.08) of the lncRNA analysis on a set of carefully-randomised pancancer SNVs.

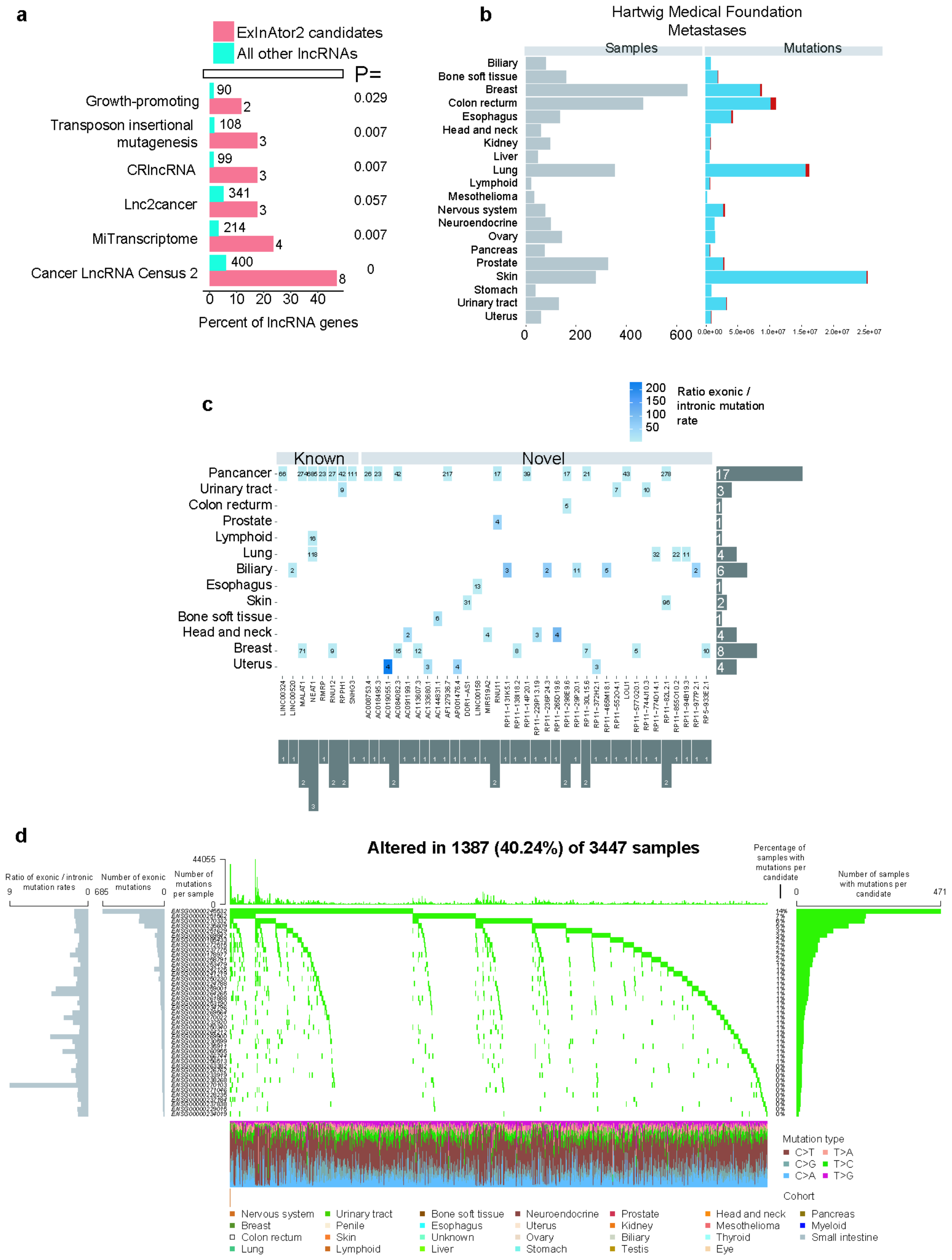
a
ExInAtor2 candidates
All other lncRNAs
P=
Growth-promoting
Transposon insertional mutagenesis
CRlncRNA
Lnc2cancer
MiTranscriptome
Cancer LncRNA Census 2
90
2
108
3
99
3
341
3
214
4
400
8
0.029
0.007
0.007
0.057
0.007
0
0 10 20 30 40
Percent of lncRNA genes
b
Hartwig Medical Foundation
Metastases
Samples
Mutations
Biliary
Bone soft tissue
Breast
Colon rectum
Esophagus
Head and neck
Kidney
Liver
Lung
Lymphoid
Mesothelioma
Nervous system
Neuroendocrine
Ovary
Pancreas
Prostate
Skin
Stomach
Urinary tract
Uterus
0 200 400 600
c
Ratio exonic / intronic mutation rate
200
150
100
50
Known
Novel
Pancancer
Urinary tract
Colon rectum
Prostate
Lymphoid
Lung
Biliary
Esophagus
Skin
Bone soft tissue
Head and neck
Breast
Uterus
LINC00324
LINC00520
MALAT1
NEAT1
RMRP
RNU12
RPPH1
SNHG3
DDR1-AS1
LINC00158
MIR519A2
RNU11
LOUI
d
Altered in 1387 (40.24%) of 3447 samples
Ratio of exonic / intronic mutation rates
Number of exonic mutations
Number of mutations per sample
Percentage of samples with mutations per candidate
Number of samples with mutations per candidate
44055
471
Mutation type
C>T
T>A
C>G
T>C
C>A
T>G
Cohort
Nervous system
Breast
Colon rectum
Lung
Urinary tract
Penile
Skin
Lymphoid
Bone soft tissue
Esophagus
Unknown
Liver
Neuroendocrine
Uterus
Ovary
Stomach
Prostate
Kidney
Biliary
Testis
Head and neck
Mesothelioma
Thyroid
Eye
Pancreas
Myeloid
Small intestine

Supplementary Figure 3

The landscape of known and novel driver lncRNAs in metastatic tumours

a) Clinical / disease properties of lncRNA drivers. Displayed are the percentage of lncRNAs that fulfill indicated criteria. LncRNAs are divided into driver-lncRNAs from PCAWG analysis (red), and all other tested lncRNAs (blue). 'Growth-promoting' refer to lncRNAs necessary for proliferation of cancer cell lines discovered by CRISPRi perturbation pooled screens of Liu et al [2]; 'Transposon insertional mutagenesis' refer to lncRNA genes orthologous to sites carrying tumour-initiating *Sleeping Beauty* insertions from mouse genome-wide screens[3]; 'CRlncRNA' – database of cancer lncrnas[4]; 'Lnc2cancer' – database of cancer lncRNAs[5]; 'MiTranscriptome' – differentially expressed lncRNAs in tumours[6]; 'Cancer LncRNA Census 2' – database of cancer lncRNAs[7]. Statistical significance was calculated by two-sided Fisher's exact test. **b)** Statistics of Hartwig Medical Foundation (HMF) tumour SNV data[8]. **c)** Candidate driver-lncRNAs discovered in HMF cohort. ExInAtor2 hits are displayed with a cutoff of FDR<0.1. Numbers inside cells represent the number of exonic SNVs. D) Oncoplot summarising lncRNA SNVs in the HMF cohort. **d) "**Oncoplot" overview of driver lncRNA analysis in HMF metastatic tumours.

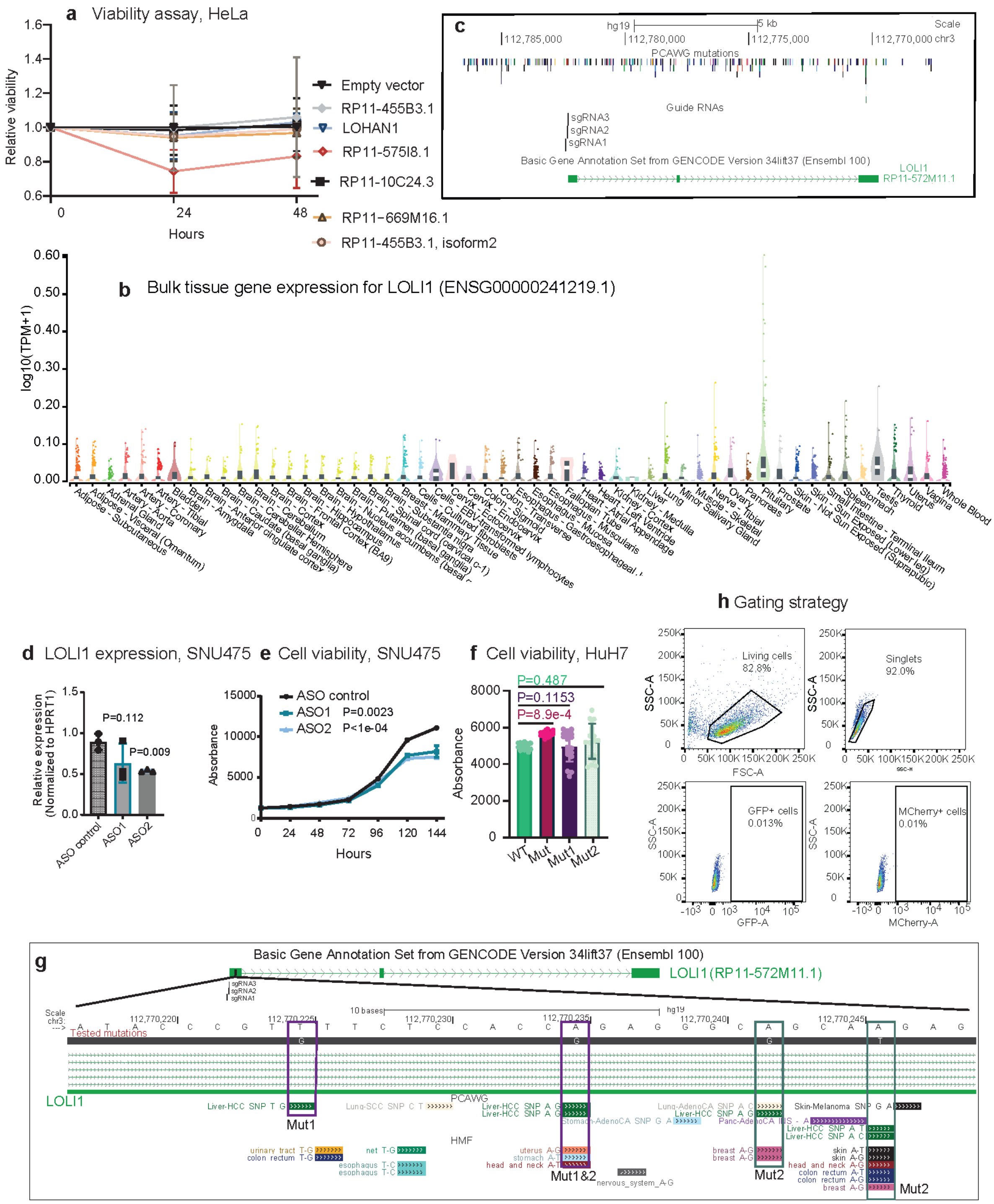
a Viability assay, HeLa
Relative viability
Hours
Empty vector
RP11-455B3.1
LOHAN1
RP11-575I8.1
RP11-10C24.3
RP11-669M16.1
RP11-455B3.1, isoform2
b Bulk tissue gene expression for LOLI1 (ENSG00000241219.1)
log10(TPM+1)
c
Guide RNAs
sgRNA3
sgRNA2
sgRNA1
PCAWG mutations
Basic Gene Annotation Set from GENCODE Version 34lift37 (Ensembl 100)
LOLI1
RP11-572M11.1
d LOLI1 expression, SNU475
Relative expression (Normalized to HPRT1)
P=0.112
P=0.009
ASO control
ASO1
ASO2
e Cell viability, SNU475
ASO control
ASO1 P=0.0023
ASO2 P<1e-04
Absorbance
Hours
f Cell viability, HuH7
P=0.487
P=0.1153
P=8.9e-4
Absorbance
WT
Mut
Mut1
Mut2
h Gating strategy
Living cells 82.8%
Singlets 92.0%
GFP+ cells 0.013%
MCherry+ cells 0.01%
SSC-A
FSC-A
GFP-A
MCherry-A
g
Basic Gene Annotation Set from GENCODE Version 34lift37 (Ensembl 100)
LOLI1(RP11-572M11.1)
Tested mutations
LOLI1
PCAWG
HMF
Mut1
Mut1&2
Mut2
Mut2

Supplementary Figure 4

Functional validations of identified driver-lncRNAs

a) Populations of plasmid-transfected cells were measured at indicated time points. Each measurement represents the mean +/- the SD of n=3 replicates. Statistical significance was estimated using one-sided Student's *t*-test with n=3 replicates at the last time point. Replicates were performed at different times (experimental replicates). **b)** Bulk tissue gene expression for LOLI1. Data was obtained from GTEXportal. **c)** LOLI1 gene locus**.** The position of the sgRNAs for CRISPRa are shown. **d)** Reverse transcription quantitative polymerase chain reaction (qRT-PCR) measurement of RNA levels in SNU476 HCC cells after transfection of control ASO, or two different ASOs targeting LOLI1. The mean +/- the SD of n=3 replicates is shown (experimental replicates). Statistical significance was estimated using one-sided Student's t-test. **e)** Populations of ASO-transfected cells were measured at indicated time points. Each measurement represents n=3 replicates (experimental replicates). Statistical significance was estimated using one-sided Student's *t*-test with n=3 independent replicates at the last time point. **f)** Cell viability of HuH7 cells 120h after transfection with plasmids expressing indicated variants of LOLI1 lncRNA. Shown are the mean and SD of n=18 replicates (experimental replicates). Statistical significance was estimated using one-sided Student's *t*-test. **g)** The genomic locus of hepatocellular carcinoma (HCC) candidate driver lncRNA LOLI1. Also shown are SNVs from PCAWG and Hartwig (HMF). The SNVs included in the mutated version of the plasmids are indicated in the violet boxes for Mut1 and green boxes for Mut2, where Mut1/2 indicate SNVs observed in two different human patients from PCAWG. **h)** Representative figure showing the gating strategy on a WT sample.

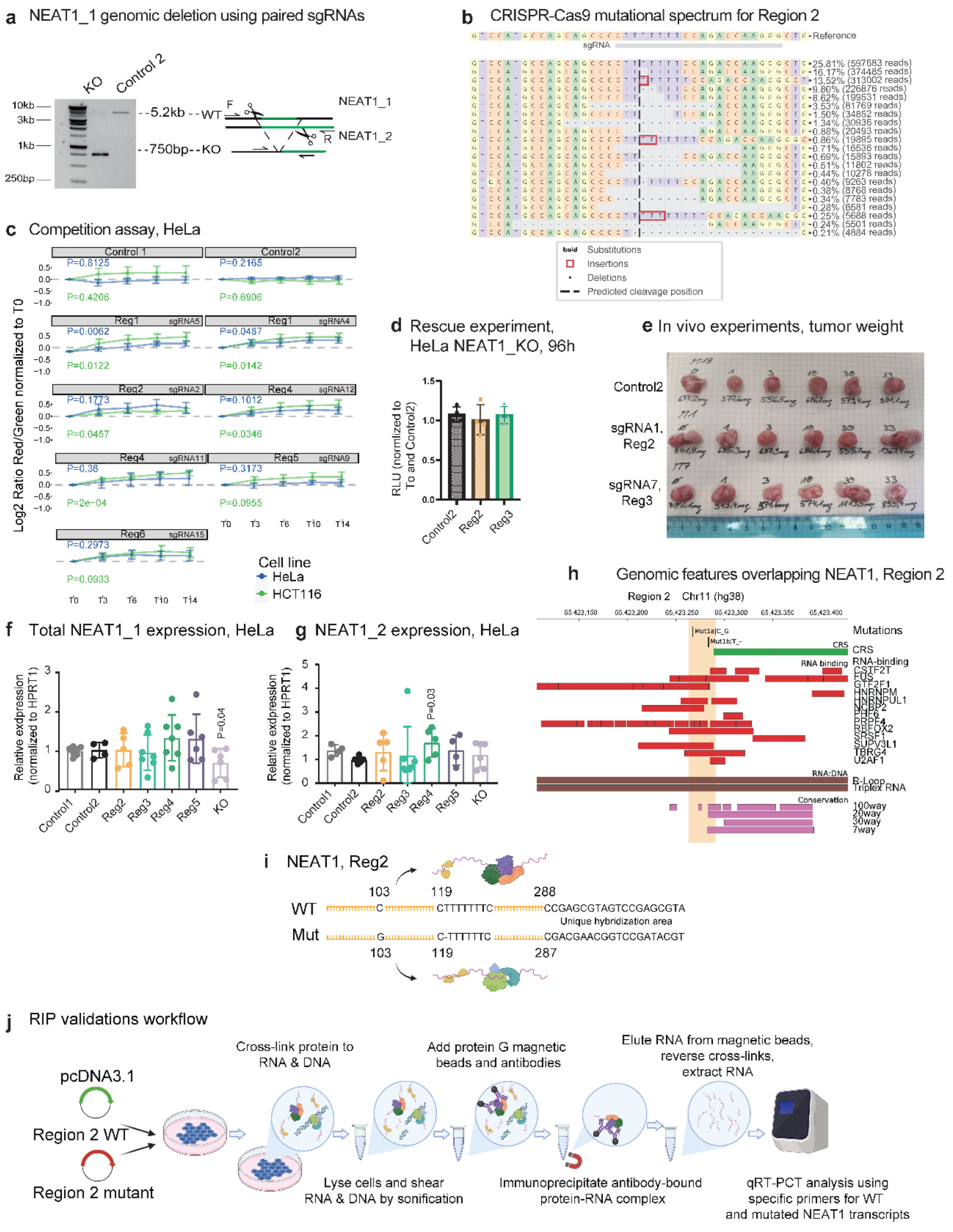

a NEAT1_1 genomic deletion using paired sgRNAs
b CRISPR-Cas9 mutational spectrum for Region 2
c Competition assay, HeLa
d Rescue experiment, HeLa NEAT1_KO, 96h
e In vivo experiments, tumor weight
f Total NEAT1_1 expression, HeLa
g NEAT1_2 expression, HeLa
h Genomic features overlapping NEAT1, Region 2
i NEAT1, Reg2
j RIP validations workflow
Cell line
HeLa
HCT116
pcDNA3.1
Region 2 WT
Region 2 mutant
Cross-link protein to RNA & DNA
Add protein G magnetic beads and antibodies
Elute RNA from magnetic beads, reverse cross-links, extract RNA
Lyse cells and shear RNA & DNA by sonification
Immunoprecipitate antibody-bound protein-RNA complex
qRT-PCT analysis using specific primers for WT and mutated NEAT1 transcripts

Supplementary Figure 5

Effect of mutations in *NEAT1* gene body

a) Genomic deletion of NEAT1_1 gene using paired sgRNAs. The panel shows agarose gel electrophoresis of PCR product (on the left) and a cartoon showing the deletion strategy (on the right) with primers amplifying the NEAT1 target region. The deletion of NEAT1_1 was checked in n=4 biological replicates. **b)** We performed deep sequencing to determine the resulting indel distribution arising from targeting Cas9 to Region 2. The reference sequence and the sgRNA are shown above. **c)** Competition assay to evaluate fitness effects of mutations. Labelled mutated (mCherry, red) and control (GFP, green) cells are mixed in equal proportions at the start of the experiment. At successive timepoints their red/green ratio is measured by flow cytometry, and this value is used to infer fitness effects. Red/green ratios for indicated mutations. "Control1/2" indicate sgRNAs targeting intergenic regions. "KO" indicates paired sgRNAs designed to delete the entire NEAT1_1 region. n=4 replicated experiments were performed, and statistical significance was estimated by linear regression model on log2 values. Replicates were performed at different times (experimental replicates). Data shows the mean value -/+ SD. **d)** Viability of NEAT1 knockout (KO) HeLa cells, in response to treatment with Control2 and two indicated NEAT1-targeting sgRNAs. Cell viability (y-axis) was measured using Cell Titer Glo assay at 96h following sgRNA delivery. n=4 biological replicates (experimental replicates). Data shows the mean value -/+ SD. **e)** Tumours extracted from 6 mice at 4 weeks post-transplantation. **f)** Normalised steady state RNA levels of NEAT1, as estimated using primers for the total NEAT1 region and **g)** using primers for NEAT1_2**.** n=4 replicated experiments were performed (experimental replicates). Statistical significance was estimated using Student's one-sided *t*-test. P-values ≥0.05 are not shown. Data shows the mean value -/+ SD. **h)** The figure shows various genomic features overlapping *NEAT1* Region 2 (highlighted). CRS: Conserved RNA Structure[9]. Red bars indicate RNA-binding proteins located by eCLIP[10]. RNA:DNA indicates experimentally-mapped chromatin interacting domains[11]. Pink bars indicate conserved regions from PhastCons. **i)** NEAT1 plasmid designs for RNA immunoprecipitation experiments. **j)** RNA immunoprecipitation experimental workflow.

Supplementary References

1. Rheinbay, E. *et al.* Analyses of non-coding somatic drivers in 2,658 cancer whole genomes. *Nature* **578**, 102–111 (2020).

2. Liu, S. J. *et al.* CRISPRi-based genome-scale identification of functional long noncoding RNA loci in human cells. *Science (1979)* **355**, aah7111 (2017).

3. Vancura, A. *et al.* Cancer LncRNA Census 2 (CLC2): an enhanced resource reveals clinical features of cancer lncRNAs. *NAR Cancer* **3**, (2021).

4. Wang, J., Zhang, X., Chen, W., Li, J. & Liu, C. CRlncRNA: a manually curated database of cancer-related long non-coding RNAs with experimental proof of functions on clinicopathological and molecular features. *BMC Med Genomics* **11**, (2018).

5. Gao, Y. *et al.* Lnc2Cancer v2.0: updated database of experimentally supported long non-coding RNAs in human cancers. *Nucleic Acids Res* **47**, D1028–D1033 (2019).

6. Iyer, M. K. *et al.* The landscape of long noncoding RNAs in the human transcriptome. *Nat Genet* **47**, 199–208 (2015).

7. Vancura, A. *et al.* Cancer LncRNA Census 2 (CLC2): an enhanced resource reveals clinical features of cancer lncRNAs. *NAR Cancer* **3**, (2021).

8. Priestley, P. *et al.* Pan-cancer whole-genome analyses of metastatic solid tumours. *Nature* **575**, 210–216 (2019).

9. Seemann, S. E. *et al.* The identification and functional annotation of RNA structures conserved in vertebrates. *Genome Res* **27**, 1371–1383 (2017).

10. Davis, C. A. *et al.* The Encyclopedia of DNA elements (ENCODE): Data portal update. *Nucleic Acids Res* **46**, D794–D801 (2018).

11. Sentürk Cetin, N. *et al.* Isolation and genome-wide characterization of cellular DNA:RNA triplex structures. *Nucleic Acids Res* **47**, 2306–2321 (2019).
