# Supplementary material for: Tumour mutations in long noncoding RNAs enhance cell fitness
Source: Nat Commun. 2023 Jun 8;14:3342. doi: 10.1038/s41467-023-39160-7 (PMC10250536; doi:10.1038/s41467-023-39160-7)
Supplement: Supplementary file 3 — Description of Additional Supplementary Files [file 41467_2023_39160_MOESM3_ESM.pdf]

File Name: Supplementary Data 1
Description: BED-format file of coordinates for low mappability and gap regions that were masked in ExInAtor2 analysis. Coordinates for genome assembly hg19.

File Name: Supplementary Data 2
Description: GENCODE gene identifiers for all lncRNAs analysed by ExInAtor2.

File Name: Supplementary Data 3
Description: The density of human common insertion sites (hCIS) per megabase of gene span for indicated lncRNAs.

File Name: Supplementary Data 4

Oligonucleotide sequences used in this study.

File Name: Supplementary Data 5

Comprehensive mass spectrometry data produced in this study.

File Name: Supplementary Data 6

Data and features of driver-lncRNAs. The data derives from the LncATLAS database (Mas-Ponte et al PMID 28386015) and further details on columns can be found therein.

File Name: Supplementary Data 7

Mutational co-occurrence results for all genes (protein-coding and lncRNA) across 15 cancer cohorts. Only statistically-significant (false-discovery adjusted $P<0.05$) results are displayed.

File Name: Supplementary Data 8

Mutational mutual-exclusivity results for all genes (protein-coding and lncRNA) across 15 cancer cohorts. Only statistically-significant (false-discovery adjusted $P<0.05$) results are displayed.

File Name: Supplementary Data 9

Source data for ezTracks analysis. Each row provides details and origin of an ENCODE eCLIP data track employed.
