# Supplementary material for: Tumour mutations in long noncoding RNAs enhance cell fitness
Source: Nat Commun. 2023 Jun 8;14:3342. doi: 10.1038/s41467-023-39160-7 (PMC10250536; doi:10.1038/s41467-023-39160-7)
Supplement: Supplementary file 13 — Reporting Summary [file 41467_2023_39160_MOESM13_ESM.pdf]

nature portfolio

Corresponding author(s): Rory Johnson and Roberta Esposito

Last updated by author(s): Apr 26, 2023

Reporting Summary

Nature Portfolio wishes to improve the reproducibility of the work that we publish. This form provides structure for consistency and transparency in reporting. For further information on Nature Portfolio policies, see our Editorial Policies and the Editorial Policy Checklist.

Statistics

For all statistical analyses, confirm that the following items are present in the figure legend, table legend, main text, or Methods section.

n/a	Confirmed	
[ ]	[x]	The exact sample size (*n*) for each experimental group/condition, given as a discrete number and unit of measurement
[ ]	[x]	A statement on whether measurements were taken from distinct samples or whether the same sample was measured repeatedly
[ ]	[x]	The statistical test(s) used AND whether they are one- or two-sided<br>*Only common tests should be described solely by name; describe more complex techniques in the Methods section.*
[x]	[ ]	A description of all covariates tested
[ ]	[x]	A description of any assumptions or corrections, such as tests of normality and adjustment for multiple comparisons
[ ]	[x]	A full description of the statistical parameters including central tendency (e.g. means) or other basic estimates (e.g. regression coefficient) AND variation (e.g. standard deviation) or associated estimates of uncertainty (e.g. confidence intervals)
[ ]	[x]	For null hypothesis testing, the test statistic (e.g. *F*, *t*, *r*) with confidence intervals, effect sizes, degrees of freedom and *P* value noted<br>*Give P values as exact values whenever suitable.*
[x]	[ ]	For Bayesian analysis, information on the choice of priors and Markov chain Monte Carlo settings
[x]	[ ]	For hierarchical and complex designs, identification of the appropriate level for tests and full reporting of outcomes
[x]	[ ]	Estimates of effect sizes (e.g. Cohen's *d*, Pearson's *r*), indicating how they were calculated

Our web collection on statistics for biologists contains articles on many of the points above.

Software and code

Policy information about availability of computer code

Data collection	Custome code is accessible at: https://github.com/gold-lab/ExInAtor2.git<br>Additionally, the code has been deposited to Zenodo (v1.0.0) [DOI: 10.5281/zenodo.7828265] and is publicly available.<br>Data and metadata were collected from International Cancer Genome Consortium (ICGC) consortium members using custom software packages designed by the ICGC Data Coordinating Centre. The general-purpose core libraries and utilities underlying this software have been released under the GPLv3 open source license as the "Overture" package and are available at https://www.overture.bio. Other data collection software used in this effort, such as ICGC-specific portal user interfaces, are available upon request to contact@overture.
Data analysis	Custome code is accessible at: https://github.com/gold-lab/ExInAtor2.git<br>The workflows executing core WGS alignment, QC and variant-calling software are packaged as executable Dockstore images and available at: https://dockstore.org/search?labels.value.keyword=pcawg&searchMode=files.<br>Individual software components are as follows: BWA-MEM v0.78.8-r455; DELLY v0.6.6; ACEseq v1.0.189; DKFZ somatic SNV workflow v1.0.132-1; Platypus v0.7.4; ascatNgs v1.5.2; BRASS v4.012; grass v1.1.6; CaVEMan v1.50; Pindel v1.5.7; ABSOLUTE/JaBbA v1.5; SvABA 2015-05-20; dRanger 2016-03-13; BreakPointer 2015-12-22; MuTect v1.1.4; MuSE v1.0rc; SMuFIN 2014-10-26; OxoG 2016-4-28; VAGrENT v2.1.2; ANNOVAR v2014Nov12; VariantBAM v2017Dec12; SNV-Merge v2017May26; SV-MERGE v2017Dec12; DKFZ v2016Dec<br>Additionally, for the data analysis we used R version 4.1.1; Mass Spectrometry data was interpreted with MaxQuant (version 1.6.14.0).

For manuscripts utilizing custom algorithms or software that are central to the research but not yet described in published literature, software must be made available to editors and reviewers. We strongly encourage code deposition in a community repository (e.g. GitHub). See the Nature Portfolio guidelines for submitting code & software for further information.

Data

Policy information about availability of data

All manuscripts must include a data availability statement. This statement should provide the following information, where applicable:

- Accession codes, unique identifiers, or web links for publicly available datasets
- A description of any restrictions on data availability
- For clinical datasets or third party data, please ensure that the statement adheres to our policy

Data availability
- Somatic mutation data:
1) Mutation WGS somatic and germline variant calls, mutational signatures, subclonal reconstructions, transcript abundance, splice calls and other core data generated by the ICGC/TCGA Pan-cancer Analysis of Whole Genomes Consortium are available for download at https://dcc.icgc.org/releases/PCAWG. Additional information on accessing the data, including raw read files, can be found at https://docs.icgc.org/pcawg/data/. In accordance with the data access policies of the ICGC and TCGA projects, most molecular, clinical and specimen data are in an open tier which does not require access approval. To access potentially identification information, such as germline alleles and underlying sequencing data, researchers will need to apply to the TCGA Data Access Committee (DAC) via dbGaP (https://dbgap.ncbi.nlm.nih.gov/aa/wga.cgi?page=login) for access to the TCGA portion of the dataset, and to the ICGC Data Access Compliance Office (DACO; http://icgc.org/daco) for the ICGC portion. In addition, to access somatic single nucleotide variants derived from TCGA donors, researchers will also need to obtain dbGaP authorization.
2) HMF data can be requested at https://www.hartwigmedicalfoundation.nl/en/data/data-acces-request/
-Somatic mutation data and clinical data:
3) The Pan-cancer Analysis of Whole Genomes Consortium (PCAWG) publicly available data used in this study for survival analysis, mutual exclusivity and co-occurrence are available in the UCSC-Xenahub database [https://xenabrowser.net/datapages/?cohort=PCAWG%20(donor%20centric)&removeHub=https%3A%2F%2Fxena.treehouse.gi.ucsc.edu%3A443] [Goldman, M. J., Craft, B., Hastie, M., Repečka, K., McDade, F., Kamath, A., ... & Haussler, D. (2020). Visualizing and interpreting cancer genomics data via the Xena platform. Nature biotechnology, 38(6), 675-678]
The remaining data are available within the Article and Supplementary Information.
Source data are provided as a Source Data file.

Research involving human participants, their data, or biological material

Policy information about studies with human participants or human data. See also policy information about sex, gender (identity/presentation), and sexual orientation and race, ethnicity and racism.

Reporting on sex and gender	We do not use sex and gender data. We collected data exclusively about mutational status.
Reporting on race, ethnicity, or other socially relevant groupings	We do not use or collect data about ethnicity.
Population characteristics	*Describe the covariate-relevant population characteristics of the human research participants (e.g. age, genotypic information, past and current diagnosis and treatment categories). If you filled out the behavioural & social sciences study design questions and have nothing to add here, write "See above."*
Recruitment	No recruitment was performed in this study
Ethics oversight	Human liver tissue was obtained from patients undergoing surgical resection for colorectal metastasis. Signed informed consent was obtained from all patients in accordance with institutional guidelines and according to study approval of the Ethics Commission of the Canton of Bern.

Note that full information on the approval of the study protocol must also be provided in the manuscript.

Field-specific reporting

Please select the one below that is the best fit for your research. If you are not sure, read the appropriate sections before making your selection.

- [x] Life sciences
- [ ] Behavioural & social sciences
- [ ] Ecological, evolutionary & environmental sciences

For a reference copy of the document with all sections, see nature.com/documents/nr-reporting-summary-flat.pdf

Life sciences study design

All studies must disclose on these points even when the disclosure is negative.

Sample size	We compiled an inventory of matched tumour/normal whole cancer genomes in the ICGC Data Coordinating Centre. Most samples came from treatment-naïve, primary cancers, but there were a small number of donors with multiple samples of primary, metastatic and/or recurrent tumours. Our inclusion criteria were: (i) matched tumour and normal specimen pair; (ii) a minimal set of clinical fields; and (iii) characterisation of tumour and normal whole genomes using Illumina HiSeq paired-end sequencing reads.<br>We collected genome data from 2,834 donors, representing all ICGC and TCGA donors that met these criteria at the time of the final data freeze in autumn 2014.<br>Regarding the wet lab experiments, a minimum of N=3 biological independent experiments was performed

Data exclusions	After quality assurance, data from 176 donors were excluded as unusable. Reasons for data exclusions included inadequate coverage, extreme bias in coverage across the genome, evidence for contamination in samples and excessive sequencing errors (for example, through 8-oxoguanine).
Replication	In order to evaluate the performance of each of the mutation-calling pipelines and determine an integration strategy, we performed a largescale<br>deep sequencing validation experiment. We selected a pilot set of 63 representative tumour/normal pairs, on which we ran the three core pipelines, together with a set of 10 additional somatic variant-calling pipelines contributed by members of the SNV Calling Working Group. Overall, the sensitivity and precision of the consensus somatic variant calls were 95% (CI90%: 88-98%) and 95% (CI90%: 71-99%) respectively for SNVs. For somatic indels, sensitivity and precision were 60% (34-72%) and 91% (73-96%) respectively. Regarding SVs, we estimate the sensitivity of the merging algorithm to be 90% for true calls generated by any one caller; precision was estimated as 97.5% - that is, 97.5% of SVs in the merged SV call-set have an associated copy number change or balanced partner rearrangement.<br>All the replication attempt were successful.
Randomization	Simulations are performed by random repositioning of mutations in exonic regions, while maintaining identical trinucleotide content.<br>For the vivo experiments, the mice were randomly allocated into the treatments groups (i.e. injection with cells previously mutated using sgRNAs targeting NEAT1 Region 2, NEAT1 Region 3 and negative control region)
Blinding	The technician performing in vivo experiments was blinded to group allocation, during data collection and analysis.

Reporting for specific materials, systems and methods

We require information from authors about some types of materials, experimental systems and methods used in many studies. Here, indicate whether each material, system or method listed is relevant to your study. If you are not sure if a list item applies to your research, read the appropriate section before selecting a response.

Materials & experimental systems

n/a	Involved in the study	
☐	☒	Antibodies
☐	☒	Eukaryotic cell lines
☒	☐	Palaeontology and archaeology
☐	☒	Animals and other organisms
☒	☐	Clinical data
☒	☐	Dual use research of concern
☒	☐	Plants

Methods

n/a	Involved in the study	
☒	☐	ChIP-seq
☐	☒	Flow cytometry
☒	☐	MRI-based neuroimaging

Antibodies

Antibodies used	Antibodies used for immunoprecipitation:<br>anti-SREK1 (Sigma, HPA037674) - 2 µg<br>anti-PQBP1 (Bethyl Laboratories, A302-802A) - 2 µg<br>Recombinant Rabbit IgG, monoclonal [EPR25A] - Isotype Control (Abcam, ab172730) - 2 µg
Validation	All the commercial antibodies are validated by the manufacturers or previous studies done by others or our laboratory

Eukaryotic cell lines

Policy information about cell lines and Sex and Gender in Research

Cell line source(s)	HeLa, HEK293T and HCT116 were a kind gift from Roderic Guigo's lab (CRG, Barcelona).<br>The MRC5-SV cells were provided by the group of Ronald Dijkmanthe (Institute of Virology and Immunology, University of Bern) and the HN5 tongue squamous cell carcinoma cells by Jeffrey E. Myers (MD Anderson) to Y. Zimmer.<br>SNU-475 were purchased from ATCC (#crl-2236)<br>HuH7 ere purchased from Cell Line Service (#300156)
Authentication	All the cell lines were authenticated using Short Tandem Repeat (STR) profiling (Microsynth Cell Line Typing)
Mycoplasma contamination	All the cell lines were tested negative for mycoplasma contamination.
Commonly misidentified lines<br>(See ICLAC register)	No commonly misidentified cell lines were used in this study

Animals and other research organisms

Policy information about studies involving animals; ARRIVE guidelines recommended for reporting animal research, and Sex and Gender in Research

Laboratory animals	NOD scid gamma mice. 6- to 8-week-old male and female mice were housed under specific pathogen-free conditions in individually ventilated cages with food and water provided ad libitum and were regularly monitored for pathogens. All animals used in the experiments were age- and sex-matched.
Wild animals	No wild animals were used in this study
Reporting on sex	Sex was not considered in this study because it was not relevant for our driver mutation study.
Field-collected samples	No field-collected samples were used in this study.
Ethics oversight	Experiments were approved by the local experimental animal committee of the Canton of Bern and performed according to Swiss laws for animal protection.

Note that full information on the approval of the study protocol must also be provided in the manuscript.

Flow Cytometry

Plots

Confirm that:

- [x] The axis labels state the marker and fluorochrome used (e.g. CD4-FITC).
- [x] The axis scales are clearly visible. Include numbers along axes only for bottom left plot of group (a 'group' is an analysis of identical markers).
- [x] All plots are contour plots with outliers or pseudocolor plots.
- [x] A numerical value for number of cells or percentage (with statistics) is provided.

Methodology

Sample preparation	Cells have been washed and resuspended in PBS 1X before the flow cytometry. Ho staining has been performed.
Instrument	BD FACS Sort LSR II
Software	BD FACSDiva 8.0.1 - FlowCore
Cell population abundance	20'000 - 50'000 cells per condition
Gating strategy	Living cells - singlets

- [x] Tick this box to confirm that a figure exemplifying the gating strategy is provided in the Supplementary Information.
